# COVID-19 Vaccination Integration: Efforts in 11 African Countries to Strengthen the Primary Health Care System

**DOI:** 10.9745/GHSP-D-23-00251

**Published:** 2024-02-20

**Authors:** Imran Mirza, Ida-Marie Ameda, Antoinette Eleonore Ba, Celestin Traore, Mekonnen Tadesse Hagos, Abdoul Aziz Gbaya, Benjamin Schreiber

**Affiliations:** a UNICEF Headquarters, New York, NY, USA.; b UNICEF Eastern and Southern Africa, Regional Office, Nairobi, Kenya.; c UNICEF Western and Central Africa, Regional Office, Dakar, Senegal.

## Abstract

Efforts to integrate COVID-19 vaccination into the primary health care system in 11 African countries have been initiated through partnerships, collaborations, and leveraging existing infrastructure.

## Key Messages

- Despite pandemic-related challenges, COVID-19 vaccination illuminated opportunities to strengthen primary health care, including community-based health systems, establish a platform for future adult vaccination, and inform pandemic preparedness efforts.
- Integration is multifaceted and multisectoral, requiring an understanding of the existing health system architecture, resources, and constraints, and can be achieved by leveraging partnerships, collaborations, and existing infrastructure.
- Including demand and awareness-generating activities and using varied communication strategies is essential for addressing hesitancy and tackling concerns and fears.

Plain language article summary available.

## BACKGROUND

The COVID-19 pandemic response, including the COVID-19 vaccine introduction and rollout, had a significant impact on essential health services, particularly immunization programs, by diverting resources and disrupting the delivery of routine health services, among others. Additionally, the pandemic response exposed the existing weaknesses within fragmented primary health care (PHC) from various causes, including long-standing neglect and insufficient investment.^1^ Low- and middle-income countries were disproportionately affected by these consequences. If resilient PHC had been in place, the human and economic toll of the crisis may have been more effectively controlled.^2^

Despite these challenges, the COVID-19 response and vaccine rollout revealed the following opportunities for strengthening PHC, making essential immunization programs more resilient, and informing pandemic preparedness: (1) holistic health worker protection, including by vaccination; (2) adult vaccination, paving the way for a life-course approach to vaccination; (3) integrated supply chain, including by upgrading cold-chain infrastructure and electronic logistics management information systems; (4) increased access to and use of improved digital tools for vaccinee registration and tracking; (5) use of tailored approaches for catch-up vaccination and reaching zero-dose communities; and (6) integration of campaigns and opportunities for integration across all PHC pillars.

The World Health Organization and UNICEF guidance “Considerations for Integrating COVID-19 Vaccination Into Immunization Programs and Primary Health Care for 2022 and Beyond” identifies ways to provide COVID-19 vaccination as an integral part of national essential immunization programs, PHC, and other health services, underpinned by key principles of sustainability, life-course approach, and leveraging of resources.^3^ A long-standing concept, integration, was embedded in the Alma-Ata Declaration (1978), envisioning the incorporation of essential health care into PHC through intersectoral action,^4^ has been pursued through approaches like Health in All Policies and the Global Immunization Vision and Strategy 2006–2015, and is a key focus of the Global Immunization Agenda 2030.^5^^–^^10^ Within the health sector, integrated delivery of immunization, family planning, and HIV-related services has been successful in many settings.^8^^,^^11^ Integration has different meanings and approaches depending on the health system objective. It is defined in global guidance as^3^:

*The partial or full adoption of COVID-19 vaccination into national immunization programme services, PHC and any other relevant health services with the overall aim of improving programme efficiency and sustainability, enhancing demand and improving user satisfaction, achieving and maintaining satisfactory coverage, and addressing inequities.*

The Strategic Advisory Group of Experts on Immunization recommended that countries should leverage the COVID-19 vaccine rollout for building resilient immunization programs and strengthening PHC.^12^

Traditionally, immunization programs have focused on children, adolescents, and women of reproductive age. The COVID-19 vaccine introduced the notion of “delivery platforms” for high-priority populations (e.g., health workers, elderly persons, people with comorbidities, and pregnant women), which provide opportunities to integrate existing vaccines for adults or new vaccines more easily into the pipeline with existing interventions (e.g., screening for noncommunicable diseases, reproductive health education, antenatal care services, and delivery of bed nets). These platforms offer an opportunity to provide a more people/family-centered approach by delivering packages of health services that better respond to users’ needs across their life course, in alignment with the goals of the Global Immunization Agenda 2030.^13^ Having those life-course immunization service platforms established and operational will serve as a critical cornerstone for quicker rollout and uptake of future adult vaccines (e.g., TB and respiratory syncytial virus) and inform pandemic preparedness efforts.

The COVID-19 vaccine helped introducing “delivery platforms” for high-priority populations, which provide opportunities to integrate new and existing vaccines with other health interventions.

This commentary aims to contribute to a collective understanding of COVID-19 vaccine integration in PHC from an assessment conducted in 11 countries (Central African Republic, Cote d’Ivoire, Eswatini, Ethiopia, Liberia, Malawi, Namibia, Nigeria, Senegal, Tanzania, and Uganda) along health system building blocks.

### Methods

Based on World Health Organization/UNICEF global guidance on COVID-19 integration, a conceptual framework was developed in October 2022 to map and understand how countries are integrating the COVID-19 vaccine into their programs along health system building blocks (Figure). In consultation with UNICEF and World Health Organization African regional offices, 11 countries were selected that had started integrating COVID-19 vaccines into different immunization activities (e.g., polio outbreak campaigns and antenatal care) and health services (e.g., routine health checks and civil services).

**FIGURE:**
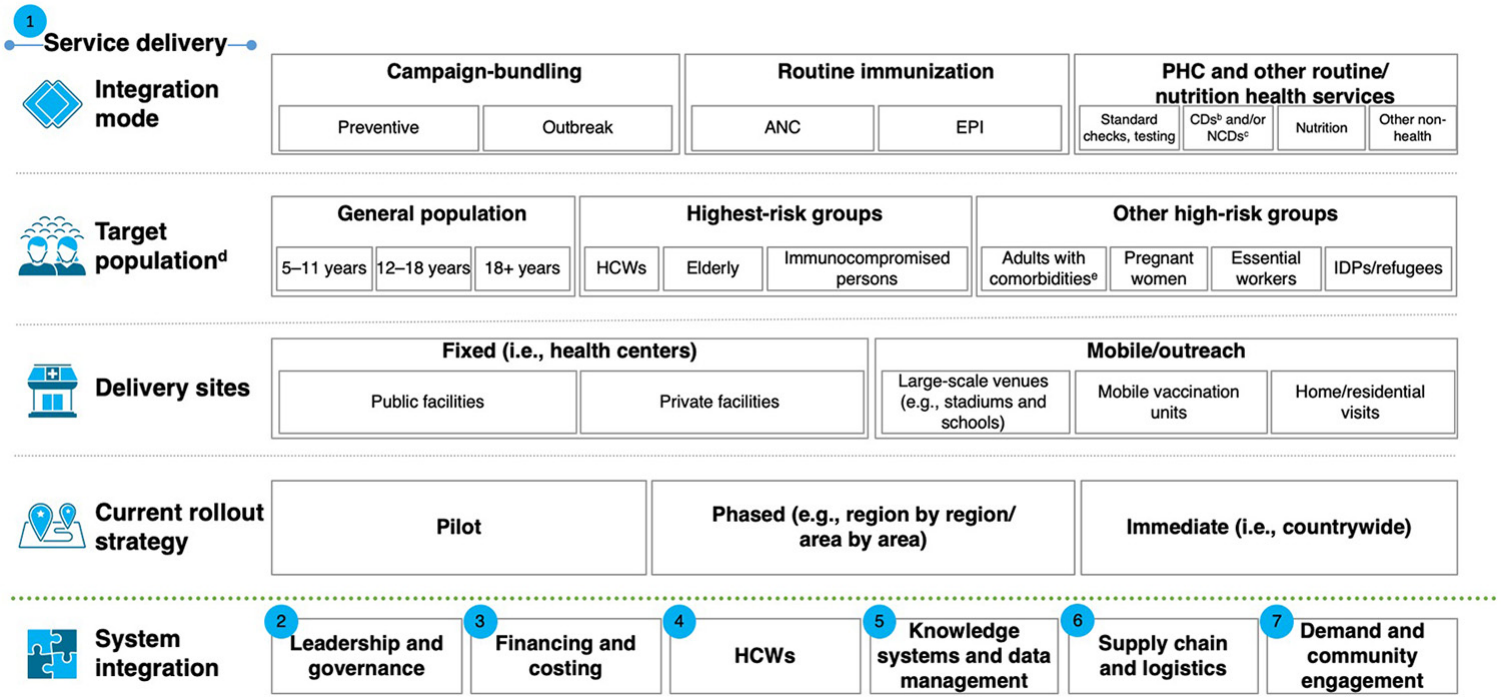
Country COVID-19 Vaccine Integration Activities Mapped Along 7 Dimensions^a^ Abbreviations: ANC, antenatal care; CD, communicable disease; EPI, Expanded Programme on Immunization; HCW, health care worker; IDP, internally displaced person; NCD, noncommunicable disease, PHC, primary health care. ^a^ Built on analysis from a desk research and key informant interviews based on World Health Organization (WHO) Health Systems Building blocks and WHO/UNICEF Guideline. (Note: the framework was adapted to allow a more granular view of service delivery and does not include pharmacovigilance and adverse events elements). ^b^ Communicable diseases such as HIV/AIDS and TB. ^c^ Noncommunicable diseases such as cancer and heart disease. ^d^ As per the WHO definition. ^e^ Including people with physical and neurological disabilities.

Information was collected through qualitative online interviews with technical teams in these countries, and a comprehensive analysis was conducted of existing literature, policy documents, and case studies to identify and categorize the success factors and challenges encountered within each domain in integrating COVID-19 vaccination into health systems.

## WHAT WERE THE SUCCESS FACTORS AND ENABLERS?

The successful integration of COVID-19 vaccination into existing health systems requires a comprehensive approach across multiple dimensions of building blocks. Multiple success factors were identified in these countries.

### Consistent High-Level Leadership and Advocacy

Consistent high-level political advocacy and support were found to be essential, with leaders emphasizing the importance of integrating COVID-19 into the health policy and strategic documents and publicly encouraging vaccine uptake among high-priority groups (e.g., in Nigeria, led by the national PHC agency). Multisectoral collaboration—including with ministries (e.g., finance and social welfare), local government, and the private sector—contributed to further expanding services and increasing demand (e.g., in Tanzania and Côte d’Ivoire).

### Leveraging of COVID-19 Financing

Countries leveraged COVID-19 funds to support integration efforts, revamp cold chain infrastructure, strengthen data systems, and deliver routine vaccinations or services alongside COVID-19 vaccination. Senegal was able to drive integration into routine immunization (RI) settings using COVID-19 funds. In Nigeria, financial working groups streamlined the effective use of funds and removed administrative barriers.

### Health Workforce Support in Line With Local Context

Health workers, who were at high risk of COVID-19 infection, were a high-priority target for vaccination and also delivered vaccines via the most appropriate opportunities across PHC. Community health workers were familiar with the local context and supported integration efforts by mapping priority groups and effectively guiding vaccination efforts in line with local preferences, needs, and beliefs.

### Integration of COVID-19 Vaccination With Other Health Services

Integration of curative and preventive health services alongside COVID-19 vaccination helped attract a wider population of high-priority groups and reduced potential stigma. Eswatini adopted a “supermarket approach” from the beginning, where communities were offered a comprehensive package of services at the same venue. Strategic organization of vaccine posts through patient triage, layout optimization, and proximity of vaccinators positively influenced vaccine perception and uptake. In Nigeria, integrated planning with RI campaigns and the implementation of a phased approach for innovative service delivery models before scaling up was also critical.

### Use of Existing Data Digital Platforms and Data Collection Tools

Identification of high-priority groups using existing databases helped in planning. National data digital health platforms integrated COVID-19 vaccination to provide information on the benefits of vaccination, site locations, appointment-making, and other services and facilitate data collection and tracking (e.g., Liberia and Malawi). Standardized checklists were incorporated into data collection tools, such as ODK, to ensure the delivery of integrated services, maintain timely reporting of vaccination data, and prevent backlogs (e.g., Nigeria).

### Strengthening of Supply Chain and Logistics

Integrated and comprehensive planning and forecasting across multiple interventions led to improved supply chain logistics, cost efficiencies, and economies of scale (e.g., Nigeria). The implementation of a reverse distribution system enabled the redistribution of vaccine stock from low-demand areas to high-demand areas and optimized waste management. Engaging the private sector, including third-party logistics firms and digital reporting consultancies, further enhanced supply chain planning, distribution, and inventory management (e.g., Mozambique).

### Demand Creation and Community Engagement

Leveraging evidence to inform and use proven behavioral and social approaches for the COVID-19 vaccine rollout helped in understanding context-specific needs and guiding integration strategies (e.g., Malawi). Establishing local platforms facilitated the sharing of real-time community feedback on perceptions and misinformation patterns, which facilitated the identification of tailored strategies. Innovative communication methods, bidirectional digital messaging platforms, and engaging celebrities and other influencers addressed mistrust and increased overall acceptance. Partnership with various groups, such as medical and allied health associations, parent teacher associations, community groups, and disease-specific support groups (for chronic diseases), ensured public buy-in and codevelopment of tailored integration strategies. For example, Liberia cocreated parental consent forms with a parent teacher association and the Ministry of Education to support school-based vaccination. Proactive engagement—including disseminating information and executing social and behavioral change activities before vaccination—generated user demand (e.g., Nigeria).

## WHAT WERE THE CHALLENGES IN ADVANCING THE INTEGRATION OF COVID-19 VACCINATION?

While integration is essential for ensuring the efficient and equitable delivery of vaccines and other services, it is a complex and multifaceted process that requires political commitment and leadership, multisectoral coordination, infrastructure development, and capacity-building of the health workforce. By identifying, understanding, and addressing challenges that exist at various levels of the health system, policymakers and health care leaders can develop targeted strategies to overcome barriers and successfully integrate COVID-19 vaccination into routine health care delivery, ultimately achieving widespread vaccine coverage and mitigating the impact of the pandemic. As a result of our assessment, the following major challenges were identified along system blocks.

### Lack of Political Commitment and Multisectoral Collaboration

In many countries, COVID-19 integration was not given high priority, primarily due to competing crises and other health priorities. This lack of political commitment and fluctuating prioritization hindered the progress of COVID-19 integration efforts. In a similar vein, the lack of an established PHC coordination platform posed a significant challenge to driving integrated services. Successful integration requires multisectoral and interdisciplinary coordination within different Ministry of Health departments (e.g., planning, preventive, and curative) and other ministries (e.g., finance, education, and social welfare). However, the willingness to establish such a cross-sectoral platform was often lacking.

### Lack of Strategic Planning to Ensure Sustainability

Integration has been primarily opportunistic, leveraging the RI platform to bundle vaccines to achieve wider coverage (e.g., integrated campaigns for measles, yellow fever vaccine, and COVID-19). Unfortunately, this approach overlooked the importance of strategic planning to ensure sustainable integration due to its primary emphasis on reaching the general population or easily accessible groups, thus missing the opportunity to strategically reach those at most risk. Furthermore, there was a prevailing tendency to view integration as a one-size-fits-all approach, disregarding the significance of context-specific needs, local factors, and tailored service delivery strategies for high-priority groups and the significance of establishing the most equitable and efficient RI and PHC systems for sustainable delivery of integrated services using COVID-19 vaccine as an entry point.

### Inadequate Financing

Inadequate financing was a significant challenge for integration efforts. Historically, funding has been allocated in a vertical, siloed manner dedicated to specific interventions or services. While there was a gradual shift toward more integrated funding approaches, a substantial portion of funding was still allocated to specific interventions. This fragmented approach hindered the comprehensive integration of services, limiting the ability to address the broader needs of communities. Although Gavi Country Delivery Support for integration is being offered to the countries, additional investments are needed to build sustainable integrated models.

Another challenge was the lack of visibility of COVID-19 vaccine-related or integration funding at the country level (e.g., at ministry partnership management units). Operational managers had limited knowledge of where funds were allocated and who was responsible for disbursing them. Donors often failed to proactively share information, leading to a lack of clarity and hindering integration planning efforts. Delay in the disbursement of funds from central repositories to local districts and implementing actors was another challenge identified.

### Overburdened Health Workforce

Health care workers (HCWs) faced significant challenges in the context of integrated settings, given pre-pandemic limited capacities and an already overwhelming workload resulting from the COVID-19 response. Expecting them to deliver a broader set of services under integrated settings put additional strain on them, which compromised the quality and effectiveness of health care services. In addition, there was limited buy-in among HCWs for integration efforts, particularly regarding the COVID-19 vaccine, which impacted the uptake of RI and broader immunization management.

### Lack of Systematic and Structured Data Collection and Management

The knowledge system and data management in integrated settings faced several challenges that need to be addressed. First, there was a lack of systematic quality data oversight and organization, including tracking capabilities, partly due to limited numbers of data staff from the national to subnational level. The absence of a formalized structure for data collection and reporting from the point of contact led to insufficient accurate data and data backlogs. Second, integrating COVID-19 data management systems into existing health information systems was a challenge, especially in areas that rely on paper-based systems. Scaling up support for vaccine stock management would also require seamless integration between different systems.

The absence of a central data hub was a concern in many countries. The lack of a comprehensive platform or system for registering vaccination data hindered the management of demand forecasting, surveillance, and follow-up activities for adverse events following immunization, as well as impeded the implementation of a national strategic plan based on accurate and timely data.

### Lack of Supply Chain and Logistics Management System

Supply chain and logistics challenges were a significant concern in integrated environments and, indeed, across health systems. The lack of appropriate physical infrastructure (due to limited investments) resulted in consistent accessibility issues. The absence of reliable transportation networks and digital connectivity further obstructed the seamless provision of integrated services to underserved regions.

Limited capability to accurately forecast vaccine demand, manage vaccine stocks, and monitor dose expiration was another significant challenge, which increased wastage and complicated vaccine management.

### Inadequate Vaccine Demand and Social Mobilization

Effective demand and community engagement are crucial for the success of integrated services, but several challenges need to be addressed. First, there is an overall vaccine hesitancy and concern among the public about the effectiveness and safety of integrating multiple vaccine antigens (pediatric and adult vaccines) through the same delivery points. Building trust and addressing these concerns through transparent and accurate communication is essential to overcome vaccine hesitancy.

Another challenge is the lack of proactive social mobilization in advance of service delivery. Often, social mobilization and communication efforts begin too close to the actual service delivery, leaving insufficient time for community engagement and education, and are not tailored to the needs and concerns of the communities.

The introduction of novel target groups in integrated services also poses a challenge. Integrating new services with population groups that are unfamiliar with them or have not received them simultaneously, such as children or adult men at the same RI service delivery point, can impact acceptance.

Despite significant challenges and given their limited resources and already strained health care systems, all assessed African countries have started implementing strategies to integrate COVID-19 vaccination into their existing PHC systems and to overcome these challenges.

## CHARTING A WAY FORWARD: WHAT PRIORITY ACTIONS ARE REQUIRED TO ADVANCE COVID-19 VACCINATION INTEGRATION INTO HEALTH SYSTEMS?

Integration of COVID-19 vaccination into existing health systems is crucial for effective vaccine delivery and ensuring equitable access (Table). The successful integration of COVID-19 vaccination into health systems requires a comprehensive approach. By implementing the identified key success factors and enablers, countries can ensure the effective integration of COVID-19 vaccination alongside routine health services, ultimately leading to improved public health outcomes. Implementing the following priority actions will enhance the integration of COVID-19 vaccination into routine health care and contribute to the resilience and effectiveness of health systems.

**TABLE.** Overarching Priority Actions Identified to Drive Further Integration in RI and PHC

Leadership and governance	Clearly outline the national strategic long-term view on the future of COVID-19 vaccination and how this translates into country-specific support programs and support activities. Establish cross-sectoral collaboration forums and coordination mechanisms within ministries and national stakeholders and broader PHC partners at national and subnational levels to expand reach and service beyond immunization teams. Define a national set of KPIs based on global KPIs to measure integration progress and effectiveness and monitor progress and reinforce accountability.
Financing and costing	Adjust in-country funds beyond COVID-19 vaccination needs to encompass integration into RI/PHC, including targeted health system strengthening measures required to lay the ground for integration. Derive root causes of funds distribution and management at all levels and address bottlenecks to ensure all COVID-19 vaccination related funds are timely deployed and utilized.
Service delivery	Develop integration plans with context-specific objectives, targeted strategies to reach priority groups, and clear governance mechanisms (like national deployment and vaccination plans), leveraging behavioral and social driver tools and arranging targeted technical assistance support for plan development where needed. Consider updating national health strategies/guidelines to position COVID-19 vaccination as part of standard patient care (e.g., integrate COVID-19 vaccination as part of standard HIV screening/treatment, NCD, and maternal, newborn, and child health clinics).
Health workforce	Map HCWs’ capacity across health care sites and services and identify gaps to facilitate sustainable COVID-19 vaccine integration. Create training curricula for HCWs at all levels on both provision and value-integrated services to increase understanding of value proposition and compliment with service-specific recommendations as needed.
Data management	Adapt data platforms (e.g., DHIS2) to enable holistic integration of relevant health information systems and tools beyond immunization, including patient-facing and program management data requirements.
Supply chain and logistics	Conduct a facility-level assessment of vaccine storage capacity, cold chain capabilities, logistics, and transportation infrastructure to identify critical bottlenecks and define readiness to integrate COVID-19 vaccine. Adapt vaccine handling and storage practices to prepare for integrated service delivery settings, including non-RI settings (e.g., TB/HIV screening, NCD clinics). Adapt global guidelines on stock waste management (e.g., multidose vials policy) to account for integration-specific considerations. Assess the impact of integration on logistics and distribution systems**/**practices (especially in non-health settings).
Demand generation and community engagement	Adapt and use proven COVID-19 behavioral and social driver tools (e.g., behavioral studies using phone surveys and focus groups) to understand context-specific behaviors, perceptions, and needs to not only optimize demand generation efforts but also implement integration strategies at all levels. Develop a balanced strategy for long-term demand creation activities between COVID-19 vaccination (especially for high-priority groups) and RI/PHC services.

Abbreviations: HCW, health care worker; KPI, key performance indicator; NCD, noncommunicable disease; PHC, primary health care; RI, routine immunization.

### Leadership and Governance

Clarifying the strategic view and value of integration is essential to guide country approaches. Developing clear guidance and integration roadmaps will help countries understand the importance and methods of integration.

Establishing or repurposing national or subnational coordination platforms and governance mechanisms will facilitate unified coordination with nonimmunization teams and expertise in managing integration processes from policy to implementation.

Conduct district-level health systems readiness assessments that focus on the needs and gaps in the health system as a whole and consider how integration could build on and enhance existing systems and capacity rather than building something new unless necessary.

### Service Delivery

COVID-19 vaccination should be positioned as standard preventive care for high-priority groups as a more comprehensive and strategic approach to integration. This will involve shifting from opportunistic campaign bundling and a one-size-fits-all approach to strategic planning and tailored integrated services designed to target specific high-priority population segments. This shift can be achieved by updating health guidelines to include COVID-19 vaccination as part of routine health care and integrating it with other interventions, such as HIV/TB screening or treatment and antenatal care. It is also important to enable bottom-up, context-specific integration activities through phased rollout strategies. This requires a thorough understanding of the local context, including cultural, socioeconomic, and geographic factors, to design and implement integration initiatives that are responsive and effective.

Defining national key performance indicators and creating a monitoring and evaluation framework will enable tracking of integration progress, with support from in-country partners and other stakeholders.

### Financing

To sustain integrated approaches, countries need to develop long-term national financing strategies that identify and allocate funding from both domestic and external sources. Adopting models like a One Plan - One Budget as implemented through the COVID-19 Vaccine Delivery Partnership can assist countries in transparent financial planning, adequate monitoring, and funds utilization for integration.^14^ In addition to Gavi Country Delivery Support funds that countries can use to operationalize and fund their integration efforts, countries can explore opportunities to leverage funding from other donors (e.g., Global Fund and United States President's Emergency Plan for AIDS Relief). These funds can be used to implement and support the integration roadmaps using the country support package, including costing of vaccine supplies, human resources, and training needs considered in integration planning and creating synergies.^15^^,^^16^

Efforts should also be made to streamline administrative processes and reduce barriers that impede the timely disbursement of funds to local districts and implementing actors. These steps are necessary to optimize the financing for integration and support the seamless implementation of integrated services.

### Health Workforce

Assessing national HCW capacity to deliver integrated services is vital for identifying gaps and facilitating smooth delivery. Leveraging volunteers or community health workers for non-injectable-related tasks (e.g., raising community demand, identifying target groups, defaulter tracing) will allow HCWs to focus on vaccine administration and reduce their workload. Other opportunities, like using preservice health workers during outreach, could also be helpful.

Health workers should undergo retraining to attain a comprehensive understanding of interconnected interventions, enabling them to proficiently administer multiple interventions concurrently. This will also emphasize the promotion of extensive community engagement to secure community support and enhance comprehension of the importance of an integrated program. Additionally, addressing HCWs’ concerns related to the COVID-19 vaccine integration through comprehensive and holistic awareness initiatives is essential for ensuring successful integration efforts. It is essential to engage with HCWs and address their concerns, provide them with accurate information, and ensure their active participation and support in integrated health care delivery.

### Health Information System

Establishing an electronic platform for data collection, reporting, and culture of data use by establishing an iterative feedback process is necessary to ensure accurate and trustworthy data. Integration of COVID-19 vaccine management systems into existing health information systems (e.g., DHIS2) should be a priority (ensuring interoperability of systems), and demand forecasting capabilities should be strengthened to optimize vaccine supply management. Additionally, developing a sophisticated central data hub and improving field reporting processes are crucial for timely and accurate data collection and analysis, supporting effective decision-making and planning in integrated settings.

### Supply Chain and Logistics

Updating the national Effective Vaccine Management assessments (vaccine storage capacity, cold chain capabilities, and logistics systems) is essential to identify areas of need and critical bottlenecks. Prioritizing investment in expanding vaccine storage and cold chain capacity, especially in underserved routine health care facilities, will improve distribution efficiency. Implementing robust inventory management systems and demand forecasting tools can help optimize stock levels and reduce wastage.

While the availability of vaccine doses is no longer a major issue, ensuring proper stock management and monitoring dose expiration using a robust supply chain management system and processes are required for the sustainability of the integrated stock of vaccines and other supplies and would facilitate achieving the goal of comprehensive immunization and other services coverage.

### Demand and Community Engagement

It is essential to develop an appropriately comprehensive approach and tailored communication strategies that address the specific needs, concerns, and cultural contexts of these high-priority groups to ensure acceptance and engagement. This involves building trust, addressing vaccine hesitancy through clear and accurate communication, providing support and training to HCWs, initiating social mobilization well in advance, and tailoring communication strategies to meet the specific needs of different target groups. By actively engaging communities, addressing concerns, and providing timely education, acceptance and uptake of integrated services can be improved, leading to better health outcomes.

Finally, enhancing transparency and information sharing among stakeholders is vital to ensure effective integration planning and implementation.

## CONCLUSION

In conclusion, the efforts to integrate COVID-19 vaccination into the PHC system in the 11 countries have been initiated through partnerships, collaborations, and leveraging existing infrastructure. While several challenges remain, the integration of COVID-19 vaccination into existing PHC systems—a complex process—requires continuous strategic planning, coordination, and monitoring for sustainable and effective vaccine delivery. With COVID-19 integration, countries have been presented with a unique opportunity to establish platforms to facilitate life-course vaccination. These platforms allow for a holistic approach to integration, recognizing that successful integration goes beyond mere economic factors and encompasses the overall well-being of high-risk populations. Utilizing COVID-19 vaccine integration investments as an opportunity to strengthen PHC and community-based systems can enhance the ability to effectively respond to future public health crises and ensures the sustained delivery of essential public health functions and the continuity of high-quality health care services. Therefore, countries must continue these efforts to strengthen their PHC systems to reach and vaccinate high- and highest-risk populations against COVID-19, as well as develop life-course vaccination platforms for future vaccines and pandemic response.

## Supplementary Material

GHSP-23-00251-article-summary-Mirza_French.pdf

GHSP-23-00251-article-summary-Mirza_English.pdf

GHSP-D-23-00251-supplement1.pdf

GHSP-23-00251-article-summary-Mirza_Portuguese.pdf

GHSP-D-23-00251-supplement2.pdf

GHSP-D-23-00251-supplement3.pdf

## Notes

**Peer Reviewed**

**First published online:** December 27, 2023.
