# Supplementary material for: COVID-19 Vaccination Integration: Efforts in 11 African Countries to Strengthen the Primary Health Care System
Source: Glob Health Sci Pract. 2024 Feb 20;12(Suppl 1):e2300251. doi: 10.9745/GHSP-D-23-00251 (PMC10948128; doi:10.9745/GHSP-D-23-00251)
Supplement: GHSP-23-00251-article-summary-Mirza_French.pdf [file GHSP-23-00251-article-summary-Mirza_French.pdf]

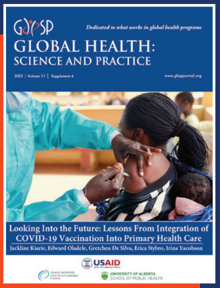

Intégration de la vaccination contre la COVID-19 : Efforts dans 11 pays africains pour renforcer le système de soins de santé primaires : Résumé de l'article

Imran Mirza, Ida-Marie Ameda, Antoinette Eleonore Ba, Celestin Traore, Mekonnen Tadesse Hagos, Abdoul Aziz Gbaya and Benjamin Schreiber

De quoi parle cet article ? L'intégration réussie de la vaccination contre la COVID-19 dans les systèmes de santé/systèmes sanitaires existants nécessite une approche qui prenne en compte les facteurs de plusieurs dimensions du système de santé/système sanitaire. Ce commentaire examine les facteurs favorables et les difficultés rencontrées dans 11 pays d'Afrique subsaharienne lors de l'intégration de la vaccination contre la COVID-19 dans le système de soins de santé primaires (SSP).

Quels étaient les résultats ? Une évaluation de l'intégration de la COVID-19 a été réalisée en 2022 dans 11 pays : République centrafricaine, Côte d'Ivoire, Eswatini, Éthiopie, Libéria, Malawi, Namibie, Nigeria, Sénégal, Tanzanie et Ouganda. Les auteurs décrivent les facteurs favorables et les défis et recommandent des actions prioritaires dans sept dimensions du système de santé/système sanitaire.

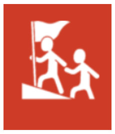

Leadership and gouvernance

Facilitateurs : Il a été essentiel de mener une action de plaidoyer et de soutien politique de haut niveau en faveur de l'intégration de la COVID-19 dans les documents politiques et stratégiques existants et d'encourager publiquement l'utilisation du vaccin.

Défis : Le manque de priorité accordée à l'intégration de la COVID-19, le manque d'engagement politique et l'absence d'une plateforme fonctionnelle de coordination des SSP préexistante ont entravé la réussite de l'intégration dans certains pays.

Actions prioritaires :

- Définir clairement les plans stratégiques nationaux pour l'avenir de la vaccination contre la COVID-19 et la valeur de l'intégration.
- Créer des mécanismes pour améliorer la collaboration et la coordination entre les secteurs.
- Définir un ensemble national d'indicateurs clés de performance pour mesurer les progrès et renforcer la responsabilité.

Points de vue des auteurs

« Avec l'intégration de la COVID-19, les pays ont eu une occasion unique de mettre en place des plateformes pour faciliter la vaccination tout au long de la vie. Ces plateformes permettent une approche holistique de l'intégration, reconnaissant qu'une intégration réussie va au-delà des simples facteurs économiques et englobe le bien-être général des populations à haut risque. »

- Ida-Marie Ameda, responsable des urgences de santé publique, Bureau régional de l'UNICEF pour l'Afrique orientale et australe

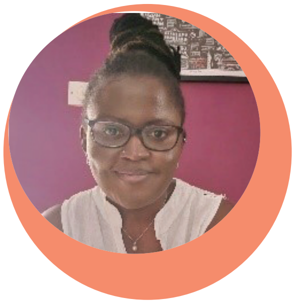

Lisez gratuitement l'article original publié dans Global Health Science and Practice à l'adresse suivante : https://doi.org/10.9745/GHSP-D-23-00251

Financement

Facilitateurs : Certains pays ont tiré parti des fonds liés à la COVID-19 pour soutenir les efforts d'intégration, réorganiser la chaîne du froid et les systèmes de données, et administrer conjointement les immunisations/vaccinations de routine contre la COVID-19.

Défis : Des financements inadéquats et cloisonnés ont entravé les efforts d'intégration dans d'autres pays.

Actions prioritaires :

- Ajuster les fonds nationaux au-delà de la vaccination contre la COVID-19 pour inclure l'intégration dans les immunisations/vaccinations de routine/SSP.
- S'attaquer aux goulets d'étranglement en matière de financement afin de garantir une distribution et une utilisation en temps voulu.

Personnel de santé

Facilitateurs : Les agents de santé, qui constituent eux-mêmes un groupe prioritaire, ont à la fois administré et identifié les meilleures occasions de distribuer des vaccins. Les agents de santé communautaire ont compris le contexte local et ont soutenu l'intégration en cartographiant les groupes prioritaires et en adaptant les orientations aux besoins locaux.

Défis : Un personnel de santé déjà surchargé a eu du mal à ajouter des activités intégrées, ce qui a compromis la qualité et l'efficacité des services de santé.

Actions prioritaires :

- Cartographier la capacité d'intégration des agents de santé et identifier les lacunes pour soutenir l'intégration des vaccins.
- Créer des programmes de formation sur la fourniture de services intégrés afin d'aider les agents de santé à comprendre la valeur de l'intégration.

Prestation de services

Facilitateurs : L'intégration d'autres services de santé et de l'immunisation/de la vaccination de routine à la vaccination contre la COVID-19 a permis d'atteindre une population plus large de groupes hautement prioritaires et a eu un effet positif sur la perception et l'utilisation du vaccin.

Défis : L'absence de planification stratégique pour assurer une intégration durable a fait manquer des occasions d'atteindre les personnes les plus exposées et de répondre aux besoins spécifiques du contexte pour atteindre les groupes prioritaires.

Actions prioritaires :

- Élaborer des plans d'intégration avec des objectifs et des stratégies spécifiques au contexte pour atteindre les groupes prioritaires.
- Mettre à jour les stratégies et les lignes directrices nationales en matière de santé afin que la vaccination contre la COVID-19 fasse partie des soins standard dans les sites de prestation de services qui ciblent les groupes hautement prioritaires.

Systèmes d’information sur la santé

Facilitateurs : L’utilisation de plateformes numériques de données nationales existantes et d’outils de collecte de données a permis d’identifier les groupes hautement prioritaires et d’établir des rapports en temps voulu.

Défis : L’absence de systèmes normalisés de collecte et de gestion des données a conduit à des données inexactes et à des arriérés de données, ainsi qu’à l’incapacité de gérer les données relatives à la demande de vaccins et à la surveillance.

Actions prioritaires :

- Adapter les plateformes de données (par exemple DHIS2) pour permettre l’intégration des données sur la santé au-delà de l’immunisation/de la vaccination.

Gestion de la chaîne d’approvisionnement

Facilitateurs : Les outils de planification et de prévision intégrés ont permis d’améliorer la logistique de la chaîne d’approvisionnement et de réaliser des économies.

Défis : Le manque d’infrastructure de la chaîne d’approvisionnement a entraîné des problèmes d’accessibilité, et la capacité limitée à prévoir la demande et les stocks de vaccins a entraîné une augmentation du gaspillage de vaccins.

Actions prioritaires :

- Évaluer la capacité de stockage des vaccins, la chaîne du froid, la logistique et les infrastructures de transport.
- Adapter les pratiques de manipulation des vaccins pour préparer la prestation de services intégrés.
- Évaluer l’impact de l’intégration sur les systèmes de logistique et de chaîne d’approvisionnement.

Création de la demande et engagement communautaire

Facilitateurs : L’utilisation de connaissance explicite pour élaborer des approches en matière de santé sociale et comportementale a aidé les programmes à comprendre les besoins locaux. Le partage des commentaires communautaires en temps réel sur les perceptions et la désinformation a permis d’adapter les stratégies aux besoins spécifiques des communautés.

Défis : L’hésitation sans précédent à l’égard du vaccin contre la COVID-19 a eu un impact sur l’intégration et l’inclusion de nouveaux groupes cibles, tandis que la mobilisation sociale et l’engagement communautaire susceptibles d’influencer l’acceptation et l’utilisation n’ont pas toujours été menés avant les campagnes.

Actions prioritaires :

- Adapter et utiliser des outils de moteurs comportementaux et sociaux de pratique éprouvée pour mieux comprendre les perceptions et les besoins afin d’améliorer la génération de la demande et de soutenir les stratégies de mise en œuvre.
- La mobilisation sociale proactive avant les campagnes permet un meilleur engagement de la communauté et est plus susceptible d’influencer l’acceptation et l’utilisation.

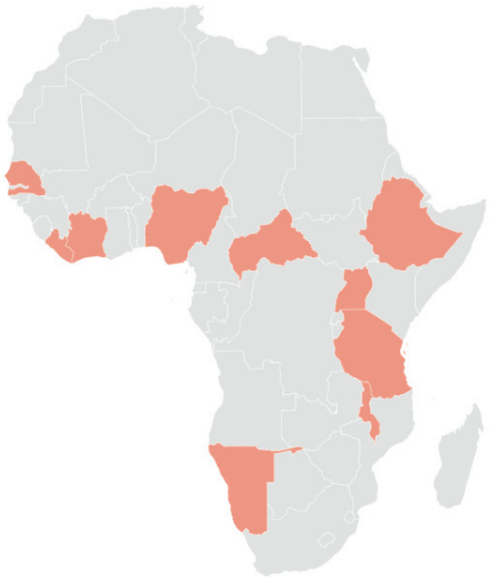

Quand et où cette évaluation a-t-elle été menée ? Les informations ont été collectées par le biais d'entretiens qualitatifs en ligne et d'une analyse de la littérature, des documents politiques et des études de cas en République centrafricaine, en Côte d'Ivoire, en Eswatini, en Éthiopie, au Liberia, au Malawi, en Namibie, au Nigeria, au Sénégal, en Tanzanie et en Ouganda en 2022.

USAID FROM THE AMERICAN PEOPLE
Knowledge SUCCESS
Ce guide a été réalisé grâce au soutien du peuple américain par l'intermédiaire de l'Agence américaine pour le développement international dans le cadre du projet Knowledge SUCCESS (Strengthening Use, Capacity, Collaboration, Exchange, Synthesis, and Sharing, accord de coopération n° 7200AA19CA00001 avec Johns Hopkins University. Knowledge SUCCESS est soutenu par le Bureau de la santé mondiale de l'USAID, le Bureau de la population et de la santé reproductive, et dirigé par le John Hopkins Centre for Communications Programs (CCP) en partenariat avec Amref Health Africa, le Centre d'économie comportementale de Busara et « FHI 360 ». Les informations fournies dans cette ressource relèvent de la seule responsabilité de Knowledge SUCCESS et ne reflètent pas nécessairement les opinions de l'USAID, du gouvernement américain ou de Johns Hopkins University.
