# Supplementary material for: COVID-19 Vaccination Integration: Efforts in 11 African Countries to Strengthen the Primary Health Care System
Source: Glob Health Sci Pract. 2024 Feb 20;12(Suppl 1):e2300251. doi: 10.9745/GHSP-D-23-00251 (PMC10948128; doi:10.9745/GHSP-D-23-00251)
Supplement: GHSP-23-00251-article-summary-Mirza_English.pdf [file GHSP-23-00251-article-summary-Mirza_English.pdf]

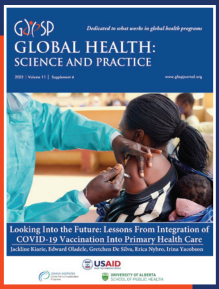

COVID-19 Vaccination Integration: Efforts in 11 African Countries to Strengthen the Primary Health Care System: Article Summary

Imran Mirza, Ida-Marie Ameda, Antoinette Eleonore Ba, Celestin Traore, Mekonnen Tadesse Hagos, Abdoul Aziz Gbaya and Benjamin Schreiber

What is this article about? The successful integration of COVID-19 vaccination into existing health systems requires an approach that considers factors across multiple health system dimensions. This commentary discusses the enablers and challenges experienced in 11 sub-Saharan African countries when integrating COVID-19 vaccination into the primary health care (PHC) system.

What were the findings? An assessment of COVID-19 integration was conducted in 2022 in 11 countries: Central African Republic, Cote d'Ivoire, Eswatini, Ethiopia, Liberia, Malawi, Namibia, Nigeria, Senegal, Tanzania, and Uganda. The authors describe the enablers and challenges and recommend priority actions across 7 health system dimensions.

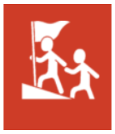

Leadership and governance

Enablers: Consistent high-level political advocacy and support for COVID-19 integration into existing policy and strategic documents and publicly encouraging vaccine uptake was essential.

Challenges: Lack of priority given to COVID-19 integration, lack of political commitment, and lack of a pre-existing functional PHC coordination platform hindered successful integration in some countries.

Priority actions:

- Clearly outline the national strategic plans for the future of COVID-19 vaccination and the value of integration.
- Create mechanisms to enhance collaboration and coordination across sectors.
- Define a national set of key performance indicators to measure progress and reinforce accountability.

Insights from the Authors

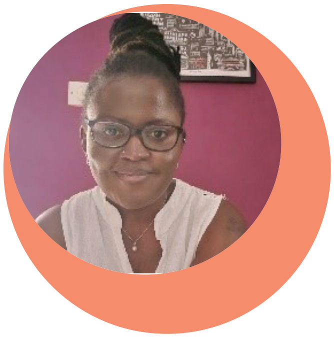

"With COVID-19 integration, countries have been presented with a unique opportunity to establish platforms to facilitate life-course vaccination. These platforms allow for a holistic approach to integration, recognizing that successful integration goes beyond mere economic factors and encompasses the overall well-being of high-risk populations."

- Ida-Marie Ameda, Public Health Emergencies Lead, UNICEF Eastern and Southern Africa Regional Office

Read the original article published in Global Health Science and Practice for free at:
https://doi.org/10.9745/GHSP-D-23-00251

Financing

Enablers: Some countries leveraged COVID-19 funds to support integration efforts, revamp cold chain and data systems, and deliver COVID-19 and routine immunizations (RIs) jointly.

Challenges: Inadequate and siloed financing hindered integration efforts in other countries.

Priority actions:

- Adjust in-country funds beyond COVID-19 vaccination to include integration into RI/PHC.
- Address bottlenecks in funding to ensure timely distribution and use.

Health workforce

Enablers: Health workers, a priority group themselves, both administered and identified the best opportunities to de-liver vaccines. Community health workers understood the local context and supported integration by mapping priority groups and adapting guidance to meet local needs.

Challenges: An already overburdened health workforce struggled to add integrated activities, compromising the quality and effectiveness of health services.

Priority actions:

- Map health care workers' capacity for integration and identify gaps to support vaccine integration.
- Create training curricula on providing integrated services to help health care workers understand the value of integration.

Service delivery

Enablers: Integrating other health services and RI with COVID-19 vaccination helped reach a wider population of high-priority groups and positively affected vaccine perception and uptake.

Challenges: Lack of strategic planning to ensure sustainable integration resulted in missed opportunities to reach those at most risk and meet context-specific needs to reach high-priority groups.

Priority actions:

- Develop integration plans with context-specific objectives and strategies to reach high-priority groups.
- Update national health strategies and guidelines to position COVID-19 vaccination as part of standard care across service delivery sites that target high priority groups.

Health information systems

Enablers: Use of existing national data digital platforms and data collection tools supported identification of high-priority groups and timely reporting.

Challenges: Lack of standard systems for data collection and management led to inaccurate data and data backlogs, as well as the inability to manage data on vaccine demand and surveillance.

Priority actions:

- Adapt data platforms (e.g., DHIS2) to enable the integration of health data beyond immunization.

Supply chain management

Enablers: Integrated planning and forecasting tools led to better supply chain logistics and cost savings.

Challenges: Lack of supply chain infrastructure resulted in accessibility issues, and the limited capability to forecast vaccine demand and stock resulted in increased vaccine wastage.

Priority actions:

- Assess vaccine storage capacity, cold chain, logistics and transportation infrastructure.
- Adapt vaccine handling practices to prepare for integrated service delivery.
- Assess the impact of integration on logistics and supply chain systems.

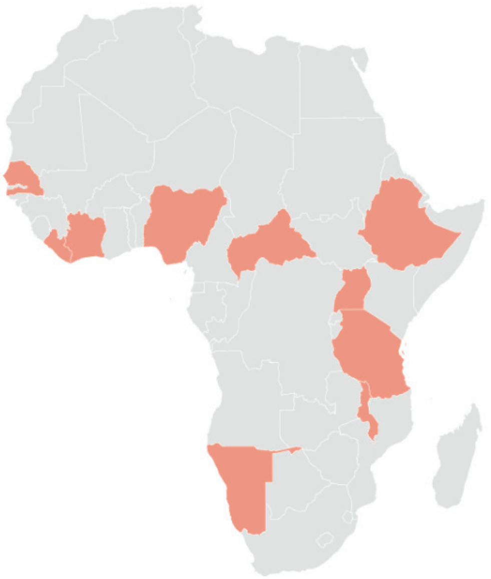

Demand creation and community engagement

Enablers: Using evidence to develop social and behavioral health approaches helped programs to understand local needs; sharing of real-time community feedback on perceptions and misinformation allowed strategies to be tailored to specific communities' needs.

Challenges: Unprecedented COVID-19 vaccine hesitancy impacted on integration and the inclusion of novel target groups, while social mobilization and community engagement that could influence acceptance and uptake was not always conducted in advance of campaigns.

Priority actions:

- Adapt and use proven behavioral and social driver tools to better understand perceptions and needs to improve demand generation and support implementation strategies. implementation strategies.
- Plan for proactive social mobilization in advance of campaigns to allow for better community engagement and increase influence, acceptance, and uptake.

When and where was this assessment conducted? Information was collected through qualitative online interviews and an analysis of literature, policy documents, and case studies in Central African Republic, Cote d'Ivoire, Eswatini, Ethiopia, Liberia, Malawi, Namibia, Nigeria, Senegal, Tanzania, and Uganda in 2022.

USAID FROM THE AMERICAN PEOPLE
Knowledge SUCCESS

This summary brief is made possible by the support of the American People through the U.S. Agency for International Development under the Knowledge SUCCESS (Strengthening Use, Capacity, Collaboration, Exchange, Synthesis, and Sharing) Project Cooperative Agreement No. 7200AA19CA00001 with the Johns Hopkins University. Knowledge SUCCESS is supported by USAID's Bureau for Global Health, Office of Population and Reproductive Health and led by the Johns Hopkins Center for Communication Programs (CCP) in partnership with Amref Health Africa, The Busara Center for Behavioral Economics (Busara), and FHI 360. The information provided in this summary brief are the sole responsibility of Knowledge SUCCESS and does not necessarily reflect the views of USAID, the U.S. Government, or the Johns Hopkins University.
