# Supplementary material for: COVID-19 Vaccination Integration: Efforts in 11 African Countries to Strengthen the Primary Health Care System
Source: Glob Health Sci Pract. 2024 Feb 20;12(Suppl 1):e2300251. doi: 10.9745/GHSP-D-23-00251 (PMC10948128; doi:10.9745/GHSP-D-23-00251)
Supplement: GHSP-23-00251-article-summary-Mirza_Portuguese.pdf [file GHSP-23-00251-article-summary-Mirza_Portuguese.pdf]

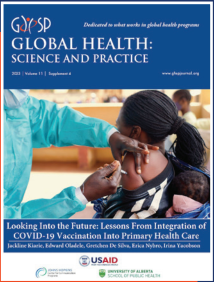

Integração da vacinação contra a COVID-19: esforços em 11 países africanos para reforçar o sistema de cuidados de saúde primários: resumo do artigo

Imran Mirza, Ida-Marie Ameda, Antoinette Eleonore Ba, Celestin Traore, Mekonnen Tadesse Hagos, Abdoul Aziz Gbaya and Benjamin Schreiber

De que trata este artigo? A integração bem-sucedida da vacinação contra a COVID-19 nos sistemas de saúde existentes requer uma abordagem que considere fatores em várias dimensões do sistema de saúde. Este comentário discute os fatores facilitadores e os desafios enfrentados em 11 países da África Subsariana ao integrar a vacinação contra a COVID-19 no sistema de cuidados de saúde primários (CSP).

Quais foram as conclusões? Em 2022, foi efetuada uma avaliação da integração da COVID-19 em 11 países: República Centro-Africana, Costa do Marfim, Essuatíni, Etiópia, Libéria, Malawi, Namíbia, Nigéria, Senegal, Tanzânia e Uganda. Os autores descrevem os fatores facilitadores e os desafios e recomendam ações prioritárias em 7 dimensões do sistema de saúde.

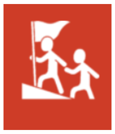

Liderança e governação

Fatores facilitadores: a defesa e o apoio políticos consistentes de alto nível para a integração da COVID-19 nos documentos políticos e estratégicos existentes e o incentivo público à adoção da vacina foram essenciais.

Desafios: a falta de prioridade dada à integração da COVID-19, a falta de empenhamento político e a falta de uma plataforma de coordenação dos CSP funcional pré-existente impediram o êxito da integração em alguns países.

Ações prioritárias:

- Definir claramente os planos estratégicos nacionais para o futuro da vacinação contra a COVID-19 e o valor da integração.
- Criar mecanismos para reforçar a colaboração e a coordenação entre setores.
- Definir um conjunto nacional de indicadores-chave de desempenho para medir os progressos e reforçar a responsabilização.

Perspetivas dos autores

"Com a integração da COVID-19, os países tiveram uma oportunidade única de estabelecer plataformas para facilitar a vacinação ao longo da vida. Estas plataformas permitem uma abordagem holística da integração, reconhecendo que uma integração bem-sucedida vai para além de meros fatores económicos e engloba o bem-estar geral das populações de alto risco."

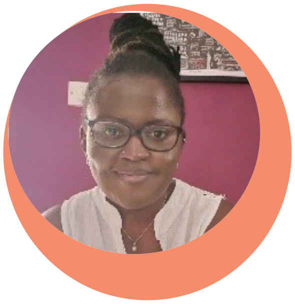

- Ida-Marie Ameda, responsável pelas emergências de saúde pública, Gabinete Regional da UNICEF para a África Oriental e Austral

Leia o artigo original publicado na Global Health Science and Practice gratuitamente em: https://doi.org/10.9745/GHSP-D-23-00251

Financiamento

Fatores facilitadores: alguns países aproveitaram os fundos da COVID-19 para apoiar os esforços de integração, renovar a cadeia de frio e os sistemas de dados, e administrar conjuntamente as imunizações de rotina (IR) e contra a COVID-19.

Desafios: um financiamento inadequado e isolado impediu os esforços de integração noutros países.

Ações prioritárias:

- Ajustar os fundos nacionais para além da vacinação contra a COVID-19, a fim de incluir a integração na IR/nos CSP.
- Resolver os estrangulamentos no financiamento para garantir uma distribuição e utilização atempadas.

Mão de obra na área da saúde

Fatores facilitadores: os profissionais do setor da saúde, eles próprios um grupo prioritário, administraram e identificaram as melhores oportunidades para administrar as vacinas. Os agentes comunitários de saúde compreenderam o contexto local e apoiaram a integração através do levantamento dos grupos prioritários e da adaptação das orientações às necessidades locais.

Desafios: uma força de trabalho no setor da saúde, já sobrecarregada, teve dificuldade em acrescentar atividades integradas, comprometendo a qualidade e a eficácia dos serviços de saúde.

Ações prioritárias:

- Mapear a capacidade de integração dos profissionais de saúde e identificar lacunas para apoiar a integração das vacinas.
- Criar programas de formação sobre a prestação de serviços integrados para ajudar os profissionais de saúde a compreender o valor da integração.

Prestação de serviçoss

Fatores facilitadores: a integração de outros serviços de saúde e da IR com a vacinação contra a COVID-19 ajudou a alcançar uma população mais vasta de grupos de elevada prioridade e afetou positivamente a perceção e a aceitação da vacina.

Desafios: a falta de planeamento estratégico para garantir uma integração sustentável resultou na perda de oportunidades de chegar às pessoas em maior risco e satisfazer as necessidades específicas do contexto para chegar a grupos de elevada prioridade.

Ações prioritárias:

- Desenvolver planos de integração com objetivos e estratégias específicos do contexto para atingir grupos de elevada prioridade.
- Atualizar as estratégias e diretrizes nacionais em matéria de saúde para posicionar a vacinação contra a COVID-19 como parte dos cuidados habituais nos locais de prestação de serviços que visam grupos altamente prioritários.

Sistemas de informação no domínio da saúde

Fatores facilitadores: a utilização de plataformas digitais de dados nacionais e de instrumentos de recolha de dados existentes apoiou a identificação de grupos de elevada prioridade e a elaboração atempada de relatórios.

Desafios: a falta de sistemas normalizados de recolha e gestão de dados conduziu a dados imprecisos e a atrasos na recolha de dados, bem como à incapacidade de gerir dados sobre a procura e a vigilância das vacinas.

Ações prioritárias:

- Adaptar as plataformas de dados (por exemplo, DHIS2) para permitir a integração de dados de saúde para além da imunização.
- Avaliar o impacto da integração nos sistemas de logística e da cadeia de abastecimento.

Gestão da cadeia de abastecimento

Fatores facilitadores: as ferramentas integradas de planeamento e previsão conduziram a uma melhor logística da cadeia de abastecimento e a poupanças de custos.

Desafios: a falta de infraestruturas da cadeia de abastecimento resultou em problemas de acessibilidade, e a capacidade limitada de prever a procura e o stock de vacinas resultou num aumento do desperdício de vacinas.

Ações prioritárias:

- Avaliar a capacidade de armazenamento de vacinas, a cadeia de frio, a logística e as infraestruturas de transporte.
- Adaptar as práticas de manuseamento das vacinas para preparar a prestação de serviços integrados.

Criação de procura e envolvimento da comunidade

Fatores facilitadores: a utilização de evidências para desenvolver abordagens de saúde social e comportamental ajudou os programas a compreender as necessidades locais; a partilha de feedback da comunidade em tempo real sobre perceções e desinformação permitiu que as estratégias fossem adaptadas às necessidades específicas das comunidades.

Desafios:: a hesitação sem precedentes em relação à vacina contra a COVID-19 teve impacto na integração e na inclusão de novos grupos-alvo, enquanto a mobilização social e o envolvimento da comunidade, que poderiam influenciar a aceitação e a adoção, nem sempre foram realizados antes das campanhas.

Ações prioritárias:

- Adaptar e utilizar ferramentas comprovadas de comportamento e de orientação social para compreender melhor as perceções e as necessidades, a fim de melhorar a criação de procura e apoiar as estratégias de implementação.
- A mobilização social proativa antes das campanhas permite um melhor envolvimento da comunidade e tem maior probabilidade de influenciar a aceitação e a adesão.

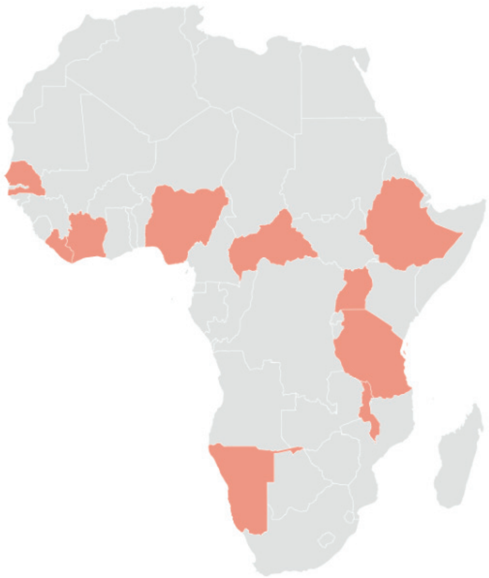

Quando e onde foi efectuada esta avaliação? A informação foi recolhida através de entrevistas qualitativas em linha e da análise de literatura, documentos políticos e estudos de caso na República Centro-Africana, Costa do Marfim, Essuatíni, Etiópia, Libéria, Malavi, Namíbia, Nigéria, Senegal, Tanzânia e Uganda em 2022.

Este guia resumido é possível graças ao apoio do povo americano através da U.S. Agency for International Development ao abrigo do Acordo de Cooperação do Projeto Knowledge SUCCESS (Strengthening Use, Capacity, Collaboration, Exchange, Synthesis, and Sharing) n.º 7200AA19CA00001 com a Universidade Johns Hopkins. O Knowledge SUCCESS é apoiado pelo Gabinete de Saúde Global, da População e Saúde Reprodutiva da USAID e dirigido pelo Johns Hopkins Center for Communication Programs (CCP) em parceria com a Amref Health Africa, The Busara Center for Behavioral Economics (Busara) e a FHI 360. As informações fornecidas neste guia resumido são da exclusiva responsabilidade da Knowledge SUCCESS e não refletem necessariamente as opiniões da USAID, do Governo dos EUA ou da Universidade Johns Hopkins.
